# Supplementary material for: A sorghum practical haplotype graph facilitates genome‐wide imputation and cost‐effective genomic prediction
Source: Plant Genome. 2020 Mar 25;13(1):e20009. doi: 10.1002/tpg2.20009 (PMC12807297; doi:10.1002/tpg2.20009)
Supplement: Supplementary file 1 [file TPG2-13-e20009-s002.pdf]

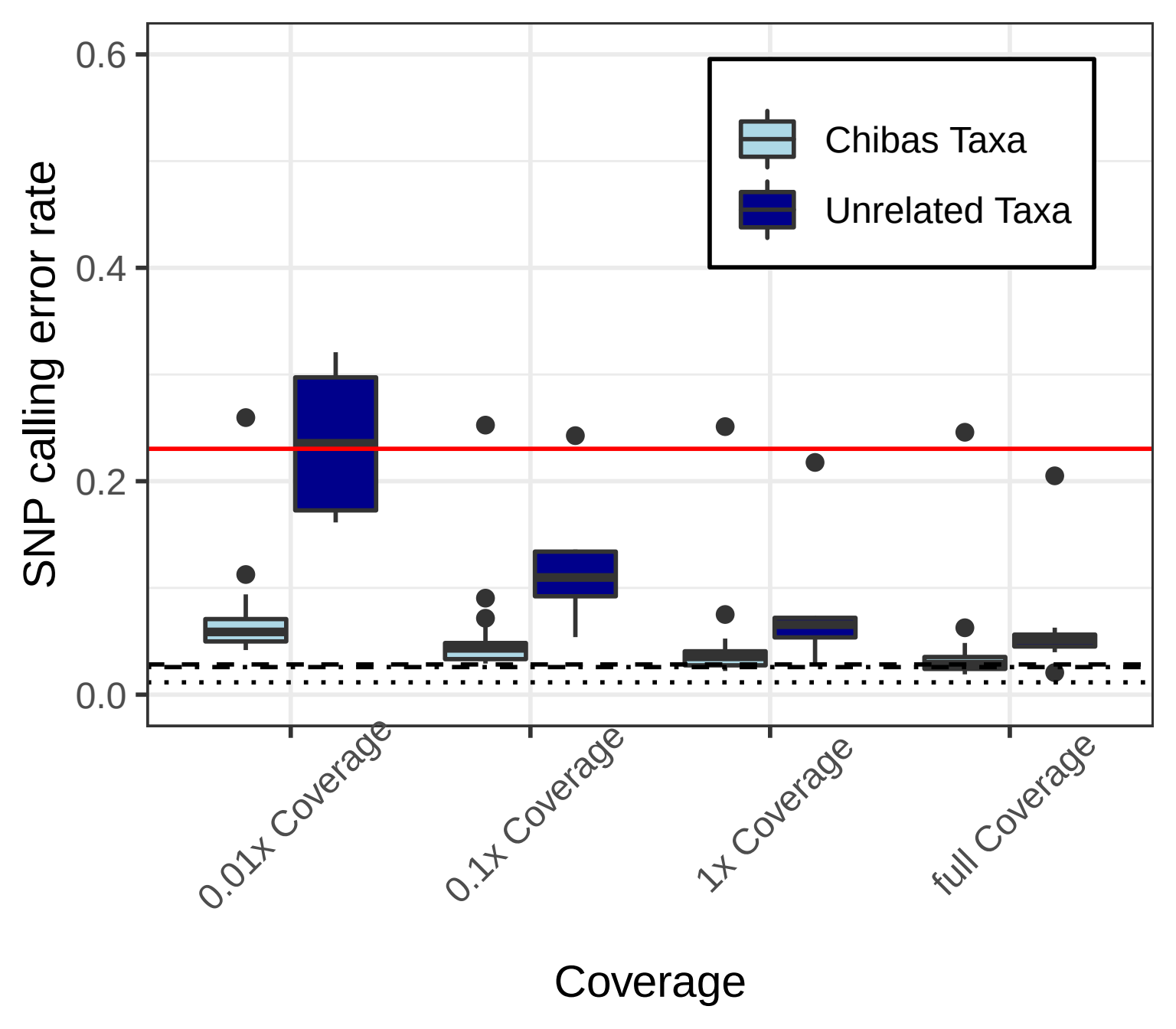

Chibas Taxa
Unrelated Taxa
SNP calling error rate
0.6
0.4
0.2
0.0
0.01x Coverage
0.1x Coverage
1x Coverage
full Coverage
Coverage
