# Supplementary material for: A sorghum practical haplotype graph facilitates genome‐wide imputation and cost‐effective genomic prediction
Source: Plant Genome. 2020 Mar 25;13(1):e20009. doi: 10.1002/tpg2.20009 (PMC12807297; doi:10.1002/tpg2.20009)
Supplement: Supplementary file 2 [file TPG2-13-e20009-s003.pdf]

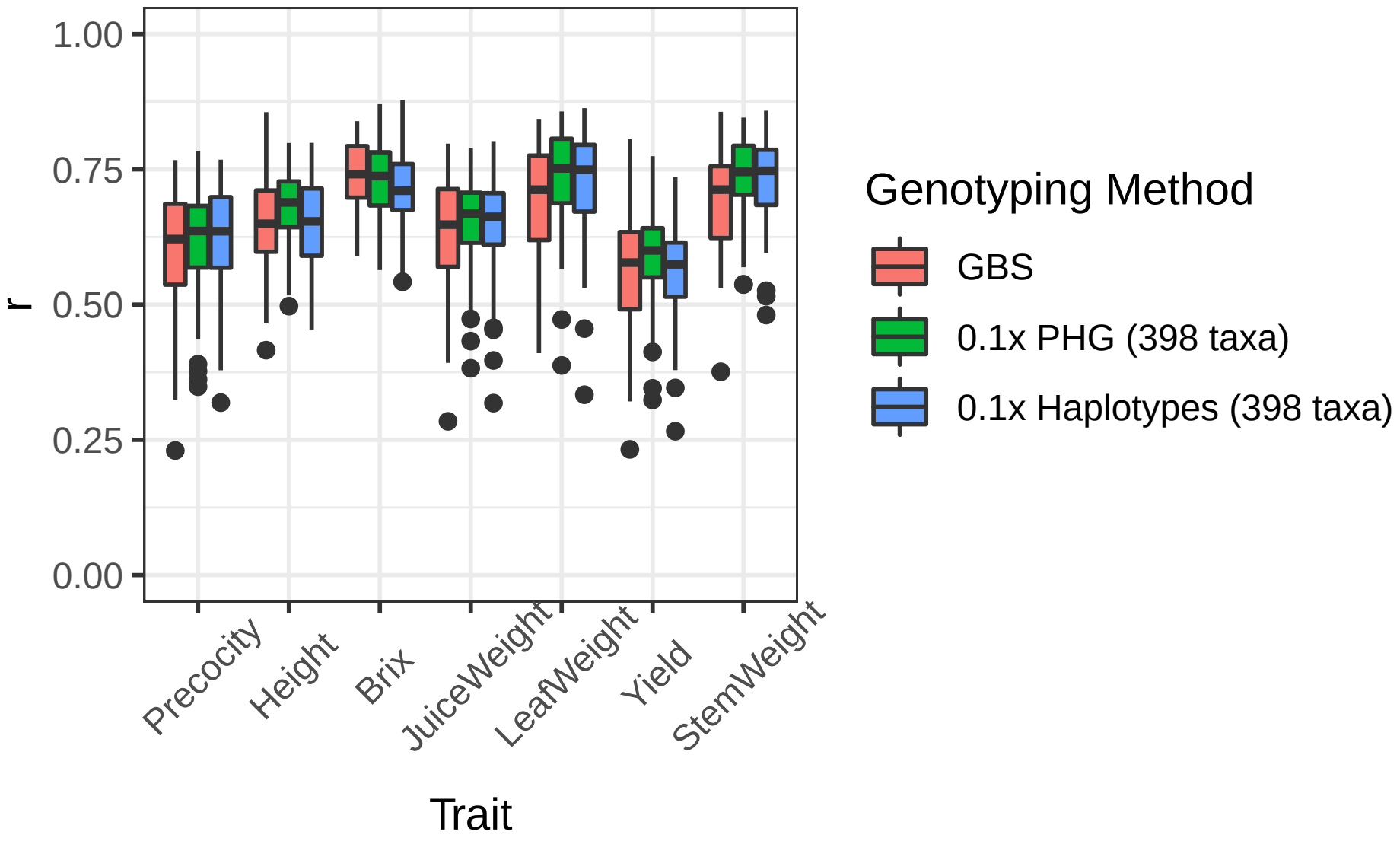

r
1.00
0.75
0.50
0.25
0.00
Precocity
Height
Brix
JuiceWeight
LeafWeight
Yield
StemWeight
Trait
Genotyping Method
GBS
0.1x PHG (398 taxa)
0.1x Haplotypes (398 taxa)
